# Supplementary material for: The causal role of circulating inflammatory markers in osteoporosis: a bidirectional Mendelian randomized study
Source: Front Immunol. 2024 Jul 18;15:1412298. doi: 10.3389/fimmu.2024.1412298 (PMC11291241; doi:10.3389/fimmu.2024.1412298)
Supplement: Supplementary file 1 [file DataSheet_1.zip › supplementary material/CIM-OSTEOPOROSIS /GCST90274805.csv_leave_one_out.pdf]

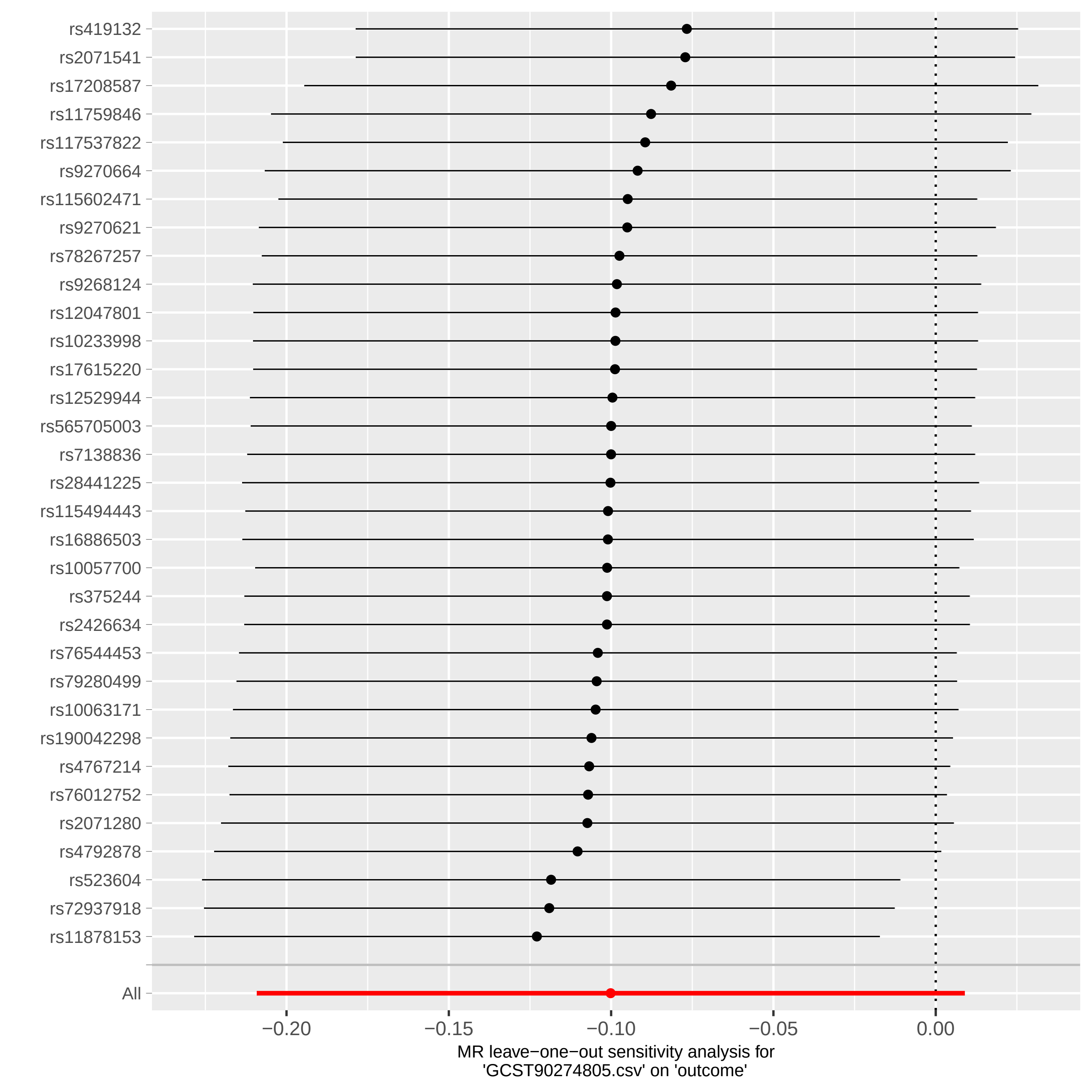
rs419132
rs2071541
rs17208587
rs11759846
rs117537822
rs9270664
rs115602471
rs9270621
rs78267257
rs9268124
rs12047801
rs10233998
rs17615220
rs12529944
rs565705003
rs7138836
rs28441225
rs115494443
rs16886503
rs10057700
rs375244
rs2426634
rs76544453
rs79280499
rs10063171
rs190042298
rs4767214
rs76012752
rs2071280
rs4792878
rs523604
rs72937918
rs11878153
All
−0.20
−0.15
−0.10
−0.05
0.00
MR leave−one−out sensitivity analysis for
'GCST90274805.csv' on 'outcome'
